# It Is in Our DNA: Bringing Electronic Health Records and Genomic Data Together for Precision Medicine

**DOI:** 10.2196/55632

**Published:** 2024-06-13

**Authors:** Alan J Robertson, Andrew J Mallett, Zornitza Stark, Clair Sullivan

**Affiliations:** 1 Faculty of Medicine University of Queensland Hertson Australia; 2 Medical Genomics Group QIMR Berghofer Medical Research Institute Brisbane Australia; 3 Queensland Digital Health Centre University of Queensland Brisbane Australia; 4 The Genomic Institute Department of Health Queensland Government Brisbane Australia; 5 Department of Renal Medicine Townsville University Hospital Townsville Australia; 6 College of Medicine and Dentistry James Cook University Townsville Australia; 7 Institute for Molecular Bioscience University of Queensland Brisbane Australia; 8 Victorian Clinical Genetics Services Murdoch Children’s Research Institute Melbourne Australia; 9 Australian Genomics Melbourne Australia; 10 University of Melbourne Melbourne Australia; 11 Centre for Health Services Research Faculty of Medicine University of Queensland Woolloongabba Australia; 12 Metro North Hospital and Health Service Department of Health Queensland Government Brisbane Australia

**Keywords:** genomics, digital health, genetics, precision medicine, genomic, genomic data, electronic health records, DNA, supports, decision-making, timeliness, diagnosis, risk reduction, electronic medical records

## Abstract

Health care is at a turning point. We are shifting from protocolized medicine to precision medicine, and digital health systems are facilitating this shift. By providing clinicians with detailed information for each patient and analytic support for decision-making at the point of care, digital health technologies are enabling a new era of precision medicine. Genomic data also provide clinicians with information that can improve the accuracy and timeliness of diagnosis, optimize prescribing, and target risk reduction strategies, all of which are key elements for precision medicine. However, genomic data are predominantly seen as diagnostic information and are not routinely integrated into the clinical workflows of electronic medical records. The use of genomic data holds significant potential for precision medicine; however, as genomic data are fundamentally different from the information collected during routine practice, special considerations are needed to use this information in a digital health setting. This paper outlines the potential of genomic data integration with electronic records, and how these data can enable precision medicine.

## Introduction

### Digital Health Care Systems Are Transforming Health Care

The adoption of electronic health records (EHRs) is transforming health care [1-4]. This digital infrastructure allows health services to store a patient’s complete medical history and collect additional observations and results in real time. Having this information in a standardized, readily accessible format provides a foundation for clinical tools to analyze these data and provide clinicians with the information to make evidence-based decisions at the point of care [1,2,4].

**Figure 1:**
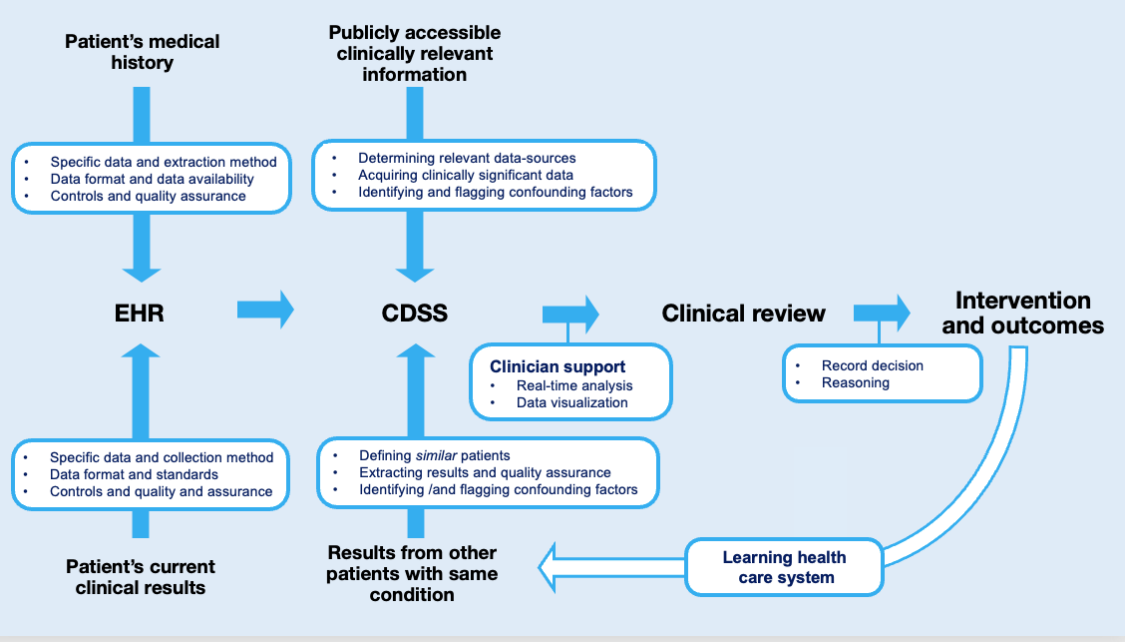
A simplified overview of a patient’s journey through a modern digitally enabled health care system, with an emphasis on the role of the EHR and CDSS. Each of the dot points linked to a solid blue arrow represents some of the specific decisions that must be made in order to integrate, analyze, and report information to clinicians. A single CDSS is not required to interact with every one of the data sources to provide clinical value but instead provide an example of some of the processes likely to occur. The white arrow represents the learning health care system, an aspirational goal for a digitally enabled health care system that uses the data collected in clinical practice as evidence for the treatment of patients afflicted with the same condition. CDSS: clinical decision support system; EHR: electronic health record.

EHRs are enabling health care to move from protocol-based medicine to precision medicine [5,6] and helping bring about the next generation of evidence-based practice. Critical to this transformation are the clinical decision support systems (CDSSs). CDSSs are electronic systems that use the information in an EHR to support the treatment of a specific disease or group of related diseases [7]. Using a patient’s data in the EHR, a CDSS processes this information in real time and presents the results to clinicians, often with the context provided by the relevant clinical guidelines [7]. The clinician is then able to filter these outputs through the lens of their clinical experience, and the nuance of the scenario, to provide an individual with a precise intervention based on their unique physiology, medical history, and current situation (Figure 1).

CDSSs are usually carefully designed by groups of experts, undergo rigorous testing, and operate within strict governance structures. As a result, CDSSs have been shown to reduce medication errors and adverse clinical events [8]. By using the information in EHRs, CDSSs allow health care systems to move past models of practice designed for paper-based systems and enable new models of care that are better able to meet the quadruple aim of health care [9,10].

One exciting model of care, enabled by EHRs and CDSSs, is learning health care systems (LHSs). An LHS uses the data collected in routine clinical practice as evidence to determine the efficacy of an intervention. These learnings can then be used to inform clinicians treating patients with the same condition. An LHS shows how using the data routinely captured by an EHR in routine practice can be used to provide value to patients, clinicians, and the broader health care system [1,2,4]; however, for many health care systems, it is an aspirational goal (Figure 1).

### Digital Health Systems Will Be Essential to Precision Medicine

Outside of LHSs, EHRs and CDSSs have the potential to facilitate a new paradigm in care—precision medicine [11,12]. Precision medicine refers to a tailored approach to care, guided by an individual’s medical history, environment, and genetic makeup [13,14]. The structured information in an EHR and the tools to contextualize and present this information to clinicians at the point of care have been used to benefit patients across a range of different areas of health [15,16]. While the capacity for digital health systems to capture and return information surrounding the patient’s medical history is well established, genomic data are not routinely incorporated into CDSSs alongside traditional clinical data sources.

### Genomic Data Are an Important Element of Precision Medicine

Genomic data are widely accepted to be a foundational component of precision medicine [13,14]. Identifying the molecular cause of a patient’s condition can lead to tailored interventions [17], a better understanding of a patient’s prognosis [18], and can help individuals make informed decisions in family planning [19]. The information in an individual’s DNA is routinely being used to provide precision clinical care across a range of different areas (Table 1). A prime example of the potential of genomic information is oncology, where genomic testing is used to identify the range of mutations acquired by an individual’s tumor, leading to tailored therapeutic interventions [20]. The management of infectious disease is another area that shows the potential of genomics in personalized medicine, as genome sequencing can be used to diagnose specific pathogen as well as determine the strain of the infectious agent as well as its antibiotic-resistance profile [21]. The information in an individual’s DNA can have tremendous potential for many different areas of precision health care. However, for many clinicians in different areas of medicine, this information is only accessible by ordering a genomic test.

**Table 1:** Clinical applications of genomics.

Application	Description	References
Diagnosis of genetic disease	While genetic testing has existed for decades, the use of next-generation sequencing has made it possible for clinicians to examine the entire genome, enabling faster and more accurate diagnosis for a broad range of rare disorders [22].	[19,22]
Disease screening and early detection	Genomic technologies can be used to identify individuals who are at a higher risk for developing certain conditions. This information can be used to manage risk and, in some cases, make interventions before the disease begins to impact the individual’s quality of life.	[19,23]
Family planning	The identification of genetic variants that place an individual at a higher risk of developing a specific condition information can be used to make informed decisions in family planning and access to reproductive technologies.	[19,23]
Cancer diagnosis, treatment, and monitoring	Cancer is a disease of the genome arising from mutations that have been acquired by an individual’s DNA [24]. By comparing the DNA from a patient’s tumor to their normal “germline” DNA, it is possible to identify the full spectrum of mutations in a tumor, including those driving disease progression. While the SHIVA study highlighted the overenthusiasm many had for this approach [25], determining the specific mutations, driving a patient’s disease, and selecting a treatment based on this information have proven to be an effective form of treatment for a range of different tumors. Moreover, monitoring a patient’s blood for the unique mutations associated with their tumor after treatment is a powerful way to monitor the progression of the disease, the effectiveness of an intervention, and if the disease is likely to reoccur [26].	[24-27]
Infectious disease diagnosis characterization	Nucleic acids are used by all living organisms. By examining patient samples, for specific nucleic acid sequences that are not from the human genome, it is possible to find sequences that are indicative of certain pathogens. The application of genome sequencing methods here provides an accurate method to detect pathogens, and in some scenarios, this approach can be used to determine the strain and specific antibiotic resistance profile of an infectious agent. As the genomes of many pathogens are significantly smaller than the human genome, it is possible to sequence large volumes of samples and screen them for pathogen DNA. The scalability of genomics in the monitoring of infectious diseases has been highlighted by the COVID-19 pandemic. Here, genomics was not only used to diagnose infection at a population scale but also to identify and track novel variants.	[21,28]
Precision treatment and pharmacogenomics	Specific genetic variants can produce molecules that behave in different ways. Some variants can completely disrupt the function of a gene, while others can change how efficiently it performs its role. As a result, certain variants can impact the way certain individuals metabolize drugs. The identification of these variants and the use of information to guide treatment can ensure that each individual receives the best intervention for their unique physiology. While only a small number of drugs are prescribed using this information, some have suggested that the metabolism of one-third of all drugs may be impacted by genetic variants.	[17,20,29-31]

### Access to the Right Genomic Data Will Enable the Realization of Precision Medicine

Population studies have revealed that each individual’s genome contains millions of different genetic variants [32]. The sheer number of variants means that it is unrealistic for a single specialist to keep track of the clinical significance of each of these variants across the range of diseases they examine. While genomic analyses would appear to be a prime candidate for the development of specialized CDSSs to support the use of genomic practice across a range of different areas of health (Table 1), CDSSs that routinely incorporate genetic information are rare [33,34]. There are likely many causes to this deficit; however, a significant factor to this can be attributed to the availability of interoperable genomic data within EHR. As a result, when many clinicians order genomic tests, the data are analyzed once, and the results are stored as a static PDF, locking the information away from future analyses.

Significant progress has been made in the development of systems to facilitate the use of genomic data in EHRs, such as clinical-grade genomic standards, file formats, and terminologies like Logical Observation Identifiers Names and Codes and Systematized Nomenclature of Medicine—Clinical Terms [35-38]. However, the adoption of these advances by EHR providers has been sluggish. As a result, EHRs are still struggling to store genomic data in a way that allows this information to be used by CDSSs. Without the capacity to access genomic data, clinicians are removed from an essential data source and will struggle to realize the full potential of precision medicine [12].

The reluctance to integrate genomic data into EHRs is likely due to a number of reasons. Some may suggest that the cause of this hesitation reflects the sheer volume and complexity of genomic data as well as the substantial amount of computer processing power and expertise required for genome analysis [39]. However, given the capacity of a VCF (variant call format) or VRS (variation representation) file to summarize the variants in a patient’s genome in a relatively potable format, the hesitancy to adopt these standards could be attributed to the complex ethical or social or legal questions surrounding genomics [12,40].

Despite these challenges, there are 2 questions that must be addressed to build a foundation to integrate genomic data into an EHR and enable genomics-empowered precision medicine: determining the right data to store and determining the right structure of these data. These questions are unlikely to have simple answers, as the answers will reflect the specific clinical questions being asked. While it is tempting to compare the virtues of exome and genome sequencing, discuss the impact of emerging technologies, or highlight the potential to bring other types of “omics” data into the EHR, these conversations are out of scope for this viewpoint. To us, it is clear that clinicians, scientists, and administrators must answer these questions together to ensure that genomic data can provide value across a range of different areas of precision medicine in their unique health service.

### Genomic Data Are New, Complex, and Different From Other Types of Health Data but Offer the Potential for New Models of Care

When determining *how* genomic data will be stored in an EHR, these conversations must address a unique attribute of genomic data—its (largely) static and unchanging nature. This attribute is typically brought up in discussions of secondary uses of genome data within the health care system [41]. However, a separate area of tremendous importance surrounds our evolving understanding of the clinical significance of a patient’s genomic data [42], as our changing understanding of the clinical relevance of a patient’s genetic data opens up new potential models of care.

The unchanging nature of a patient’s DNA and a rapidly changing understanding of the importance of that data mean that if a patient did not receive a molecular diagnosis after genomic testing, reanalyzing the same information at a later date with the context of new discoveries and new techniques can produce new molecular diagnoses [43-45]. While discovery and changing understandings are not unique to genomics, in contrast to other fields, the *rate* and *volume* at which new genomic information is accumulating is so extraordinary that reinterpreting existing genomic data with the context provided by new discoveries is known to increase diagnostic yields [42].

Special considerations will be needed to harness the levels of change associated with genomic data when designing genomics-enabled EHRs and CDSSs. Moreover, they highlight the need for these digital solutions to alert laboratories and clinicians when clinically important information has changed and robust systems in place for clinicians and laboratories to be empowered to use this information (Textbox 1).

**A clinical vignette.**

To contextualize the static nature of genome data and our changing understanding of that data, a patient aged 9 years may present to the clinic with the hallmark signs of a metabolic disorder. However, genomic testing might not confidently identify a causative pathogenic variant. Suppose the patient’s existing genomic data are routinely reanalyzed when the patient reaches the age of 14 years. In that case, clinicians are able to take advantage of all the genes found to be associated with metabolism that have occurred in the last 5 years. This information could be used to inform the patient’s treatment or potentially slow their decline. This example also highlights the potential for a “push” style approach, in which the clinician is alerted each time a gene associated with metabolism is discovered—ensuring that the patient can benefit from this new information as soon as it occurs.

### Moving From Prescriptive to Precision Medicine

While there is still work to be done, the eventual widespread adoption of genomic-enabled EHRs will facilitate the move from a traditional, prescriptive approach to medicine to personalized models of care. However, this will require a change in the way we approach genomic testing.

Currently, genomic tests resemble a “pull-based” approach. In this approach, only the genes of interest are analyzed, and the additional information needed to contextualize a patient’s genetic variants is “pulled” from the literature or analysis resources once. While there is a movement away from this philosophy, the singular, request nature of this approach prevents patients and clinicians from benefiting from our rapidly evolving understanding of genetic variants.

An alternative approach would be to perform genome sequencing once and store this information with the view that it will be used across the range of interactions an individual would have with the health system throughout their lifetime (Table 2). This will be facilitated by storing the data in structured, secure, interoperable formats, with the assumption that these data will be aligned to newer reference genomes, analyzed with different variant callers, and compared to constantly evolving virtual gene panels. While the raw genomic data might not need to be directly accessible in the EHR, reliable access to genome data will support every future interaction with a precision medicine–enabled health care system.

In this model, a CDSS could be designed around a “push” model. In the event of an inconclusive test, changes in the amount of information associated with the condition can be automatically monitored, and when it passes a threshold, the EHR can alert both the patient and the clinician to the potential for reanalysis. Patients who receive a molecular diagnosis from genomic testing could still benefit from continued monitoring by a CDSS. For example, the CDSS could highlight novel treatment interventions based on new information, such as new, targeted pharmacogenomic recommendations and potential clinical trial opportunities.

Key to this approach is the accessibility of genomic data for CDSSs. To give CDSSs access in a safe and transparent manner, there are significant challenges to overcome. Some of these challenges will be addressed from a bioinformatics perspective; however, others will require a clinical or health informatics solution, and some others still will require a policy or multidisciplinary approach.

**Table 2:** Moving to a model of genomics-enabled precision medicine.

Activity	Genetic+genomic testing	
	Traditional practice	A potential model of genomics-enabled care
Generation of sequence data	DNA from the genes associated with the condition is sequenced when a test is ordered	Individual’s whole genome sequence is available from a prior interaction with the health care system. A CDSS^a^ recommends if there is a benefit to generate complementary sequence data (eg, long read, transcriptomic, cell-free).
Analysis and interpretation of genetic data	Variants within the sequenced DNA are determined The clinical significance of the variants is accessed	A CDSS accesses the specific genes currently associated with condition from multiple high-quality, peer-reviewed resources. A CDSS recommends if genome data should be aligned to a new reference genome or use updated variant detection methods. Variants within the selected genes are determined. The clinical significance of the variants is accessed.
Clinical decisions and reporting	Clinician synthesizes genetic results, patient’s history, and clinical experience to make decision A clinical report is generated Report is uploaded to the EHR^b^ as a PDF	Clinician synthesizes genetic results, patient’s history, and CDSS recommendations through the lens of their clinical experience to make decision. The CDSS interacts with LIMs^c^ and identifies any potential pharmacogenomic interventions or potential interactions. A clinical report is generated. Findings reported to patient and other clinicians (secure portal+PDF). Report findings to EHR. Flag that the test was successful or inconclusive. If successful, share causative variants with public repositories and related individuals. Make results accessible to other clinicians treating the individual (where appropriate). If inconclusive, flag candidate variants of uncertain significance for automatic monitoring, monitor information associated with disease, and determine when the individual should be reanalyzed.
Data storage	Raw sequence data and results stored in the laboratory system Note: external collaborators do not always provide raw-sequence data	Store raw sequencing data, processed results, and variant interpretations in laboratory LIMs. Store all clinically significant (and potentially significant) variants in EHR. Ensure all information is in a standardized interoperable and time-stamped format (ie, GA4GH or eMerge).

^a^CDSS: clinical decision support system.

^b^EHR: electronic health record.

^c^LIM: Laboratory Information Management System.

## Conclusions

The clinical potential of integrating genomics information with the range of clinically relevant data collected by an EHR has been long recognized as an important element for precision medicine [46]. However, the slow adoption of the standards needed to capture and use genomic data alongside the other information in the EHR is preventing the realization of this potential. Moreover, as genomic data associated with unique attributes are so different from other health care data, special considerations are needed to harness this potential when designing the systems. As many health care systems are revising their digital health strategies, there is an opportunity to address this oversight and guide the development of EHRs that are committed to determining and incorporating the right kinds of genomic data for their unique needs.

EHRs that have been designed to accommodate the unique attributes of genomic information will benefit patients, clinicians, and health services. These EHRs will enable the production of disease-specific, genomic-enabled CDSS applications, allow more clinicians to use genomic data in practice, and collect information that can be used to better characterize relationships between genotype and phenotype. Together these systems will support precision medicine, and also provide a framework to capture the efficacy of genomically informed treatments, for a next-generation, genomics-empowered LHS.

## Abbreviations

CDSS: clinical decision support system
EHR: electronic health record
LHS: learning health care system
